# Risk factors of cholangiocarcinoma in areas not endemic for liver fluke infection

**DOI:** 10.2478/abm-2024-0028

**Published:** 2024-10-31

**Authors:** Tongluk Teerasarntipan, Pawat Phuensan, Chonlada Phathong, Somchai Pinlaor, Parit Mekaroonkamol, Roongruedee Chaiteerakij

**Affiliations:** Division of Gastroenterology, Department of Medicine, Faculty of Medicine, Chulalongkorn University, Bangkok 10330, Thailand; Department of Medicine, Faculty of Medicine, Hospital and Ambulatory Medicine Unit, Chulalongkorn University, Bangkok 10330, Thailand; Department of Parasitology and Cholangiocarcinoma Research Institute, Faculty of Medicine, Khon Kaen University, Khon Kaen 40002, Thailand; Division of Gastroenterology, Center of Excellence for Innovation and Endoscopy in Gastrointestinal Oncology, Faculty of Medicine, Chulalongkorn University, Bangkok 10330, Thailand

**Keywords:** bile duct cancer, cholangiocarcinoma, liver fluke, *Opisthorchis viverrini*, risk factor

## Abstract

**Background:**

Thailand has the world’s highest prevalence of cholangiocarcinoma (CCA), especially in the endemic area of liver fluke *Opisthorchis viverrini* infection. However, other regions of Thailand still have relatively high CCA prevalence.

**Objectives:**

We aimed to determine CCA risk factors in areas not endemic for OV infection.

**Methods:**

A case-–control study was performed at a referral center during December 2016–December 2017. We collected blood samples and information from CCA patients and identified them as cases. The control group comprised patients who visited a gastrointestinal clinic for colorectal cancer screening colonoscopy. Logistic regression analysis was used to determine risk factors for CCA.

**Results:**

Of 138 participants, *O. viverrini* infection rate was higher in the case than in the control group (57.1% vs. 36.1%, *P* = 0.023). Male, *O. viverrini* infection, smoking, alcohol consumption, and biliary tract diseases were independent risk factors, whereas diabetes, obesity, and cirrhosis were not associated with CCA. By age and sex-adjusted analysis, chronic biliary tract diseases, especially choledochal cysts and smoking, were risk factors for CCA, with adjusted odds ratio (aOR) of 12.7 (95% confidence interval [CI]: 1.4–116.9) and 3.8 (95% CI: 1.3–11.8), respectively, while *O. viverrini* infection became insignificant risk for CCA (aOR 1.8, 95% CI: 0.8–4.1).

**Conclusions:**

In contrast with endemic areas for *O. viverrini* infection, chronic biliary tract diseases and smoking are major risk factors, whereas *O. viverrini* infection has trivial contribution to the development of CCA.

Cholangiocarcinoma (CCA), a cancer of bile duct epithelium, is found worldwide with varied incidence among different areas (2.8–7.5 per 100,000 population among Asian countries and 1.2–3.4 per 100,000 population among Western countries) [1, 2]. Thailand has by far the highest incidence of CCA in the world, with reported frequencies ranging from 38.6 to 83.4 patients per 100,000 population [3, 4]. Geographically, Thailand is divided into 6 regions: Northern, Northeastern, Western, Southern, Eastern, and Central, with distinctive lifestyles and cultures. The CCA incidence varies according to geographical regions, with the highest incidence in Northeast Thailand, corresponding to the varied prevalence of *Opisthorchis viverrini* infection, a well-recognized etiology of CCA [1].

A nationwide study in Thailand in 2017 reported that even in non-endemic areas of *O. viverrini* infection, such as the central region including Bangkok, with *O. viverrini* prevalence of only 16.7% [5], had a relatively high prevalence of CCA, that is, 6.0–9.2 patients per 100,000 population, respectively [1]. Because the prevalence of CCA in non-*O. viverrini* endemic area in Thailand is still very high in comparison to the global average [1], this leads to the question of whether there are more influential etiologies of CCA other than *O. viverrini* infection in this group of patients.

Primary sclerosing cholangitis (PSC), one of the major causes of CCA among westerners, is rare in Southeast Asians [6]. Hence, some factors, such as chronic liver diseases, metabolic-related disorders (type 2 diabetes mellitus [T2DM], obesity), smoking, or alcohol consumption that were previously recognized as minor contributing factors for CCA [2, 7], might have a greater impact on cholangiocarcinogenesis in areas not endemic for *O. viverrini* infection and PSC. This study, therefore, aimed to determine whether these factors contribute to CCA in areas not endemic for *O. viverrini* infection.

## Materials and methods

Based on Kelsey’s formula for an unmatched case–control study, we used information from a population-based study in Thailand [7]; 40% of control exposed, ratio of case per control of 1.0, odd ratio of 3.09, a total of 164 participants needed to be recruited. The study followed the principles of the contemporary revision of the Declaration of Helsinki. This study was registered to the Thai clinical trial registry (TCTR No. 20200709005) and was approved by the Institutional Review Board of the Faculty of Medicine, Chulalongkorn University (IRB No. 568/59). All participants gave their informed consent prior to study enrollment.

We included all Thai patients aged ≥18 years who were newly diagnosed with CCA at our tertiary referral center during December 2016–December 2017 (case group). All patients had histopathology-confirmed CCA with clinical features of either obstructive jaundice, liver mass, and/or high serum CA19-9 of >100 U/mL, together with radiologic evidence of bile duct malignancy by computed tomography or magnetic resonance imaging. The pathological specimens were obtained by one of the following: surgical resection, endoscopic ultrasound-guided core needle biopsy, percutaneous needle biopsy, intraductal biopsy, or intraductal brush. Exclusion criteria were mixed hepatocholangiocarcinoma and having other concurrent malignancy. Asymptomatic individuals who visited the gastrointestinal clinic for colorectal cancer screening were consecutively enrolled as controls. Exclusion criteria for the control group was having concurrent, either active or inactive, malignancy. Patients who had their hometown or were nurtured in an endemic area of liver fluke infection were also excluded because they were at risk of past *O. viverrini* infection which might had some long-term carcinogenic effects. Demographic data and laboratory results were obtained from medical records. Clinical information was obtained using questionnaire and personal interview by one investigator (PP) who was not involved in statistical analysis and manuscript preparation.

We diagnosed *O. viverrini* infection using serum antibody detection by enzyme-linked immunosorbent assays (ELISA) technique. During sample collection process, blood samples for *O. viverrini* infection test from each participant were stored at −80°C. After study recruitment was completed, all blood samples were sent to detect serum IgG antibody to crude extract of *O. viverrini* antigen using ELISA technique (Dynatech, MR500, US) [8]. Blood tests were performed within 7 days after study enrollment.

We investigated other potential CCA risk factors [1,2,3, 9] including chronic biliary tract disorders (PSC, choledochal cyst and hepatolithiasis), chronic viral hepatitis B (HBV) and C (HCV) infection, cirrhosis, non-alcoholic fatty liver disease (NAFLD), T2DM, obesity, alcohol consumption, smoking, and family history of cancer. Current address, defined as the main region of living during the past 10 years, was classified according to the geographical regions of Thailand. Obesity was defined as body mass index (BMI) ≥25 kg/m2 according to the Asia-Pacific obesity classification. Family history of cancer, alcohol drinking, and smoking was recorded by patient interviews or evidence from medical records. Significant alcohol drinking was defined as >60 g and >40 g of alcohol intake per week in men and women, respectively, for >10 years. A significant history of smoking was defined as smoking >5 pack-year. HBV and HCV infection were defined as positive for hepatitis B surface antigen (HBsAg) and detectable HCV RNA in blood, respectively. Cirrhosis was diagnosed by radiologic evidence of a nodular liver, caudate lobe hypertrophy, or portal hypertension. NAFLD was diagnosed by histopathology or evidence of fatty infiltration on radiologic imaging or controlled attenuated parameters obtained from transient elastrography (FibroScan^®^, Echosens), no history of significant alcohol drinking, and exclusion of other chronic liver diseases. T2DM was defined as fasting plasma glucose ≥126 mg/dL or currently taking anti-diabetic agents Hepatolithiasis was diagnosed by radiologic evidence. A diagnosis of the above diseases in physician’s notes was also acceptable as evidence of disease.

### Statistical analysis

Categorical variables were described as counts and percentages, and compared using the Fisher’s exact test. Continuous variables were displayed as means (±standard deviation [SD]), and compared using independent sample *t* test or the Mann–Whitney (Wilcoxon rank) test for normal and non-normal distributed data, respectively. Association between each variable and CCA was initially assessed by univariate logistic regression analysis. Significant parameters were subsequently included in the multivariate model. Odds ratio (OR) along with 95% confidence interval (95% CI) were calculated using logistic regression analysis and Mantel–Haenszel methods for estimating the direction and magnitude of association between each variable and CCA. All tests were 2-sided, and the *P*-value for the significance level was <0.05.

## Results

### Baseline characteristics

There were 164 patients who were initially recruited. We excluded 26 participants who had current address from *O. viverrini* endemic region, thus a total of 138 participants enrolled the study (57 CCA patients and 81 controls). Regarding CCA subtypes, 19 (33.3%), 27 (47.4%), and 11 (19.3%) cases had intrahepatic CCA (iCCA), perihilar CCA (pCCA), and distal CCA (dCCA), respectively. A mean age of the entire cohort was 61.7 + 10.0 years, which was similar to the mean age of both the case and control groups (*P* = 0.841). Patients with CCA had a higher proportion of male gender (65.7% vs. 44.6%, *P* = 0.026). Most participants (n = 99, 71.7%) were resided in Bangkok (**Table 1**). Regarding ethnics for patients living in Bangkok, 74.7% were Bangkokians, whereas 10.1% were Northeastern patients.

**Table 1.** Baseline demographic characteristics

**Variables**	**Overall (n = 138)**	**CCA patients (n = 57)**	**Control participants (n = 81)**	***P***
Age (mean ± SD), year	61.7 ± 10.0	61.5 ± 12.6	61.8 ± 7.7	0.841
Male gender	70 (50.7%)	35 (65.7%)	35 (44.6%)	0.026
Address				
Bangkok	99 (71.7%)	30 (52.6%)	69 (85.2%)	<0.001
East	19 (13.8%)	13 (22.8%)	6 (7.4%)	0.048
West	9 (6.5%)	7 (12.3%)	2 (2.5%)	0.034
Other regions	11 (8.0%)	7 (12.3%)	4 (5.0%)	0.170
BMI (mean ± SD), kg/m^2^	23.9 ± 3.6	23.8 ± 3.6	24.0 ± 3.6	0.765
OV infection	60 (43.5%)	31 (57.1%)	29 (36.1%)	0.023
Unhealthy lifestyles				
Smoking	32 (23.3%)	23 (40.4%)	9 (11.1%)	<0.001
Alcohol consumption	15 (10.9%)	14 (24.6%)	1 (1.2%)	<0.001
Chronic biliary tract disorders	10 (6.5%)	8 (14.0%)	1 (1.2%)	<0.001
Hepatolithiasis	9 (6.5%)	7 (12.3%)	1 (1.2%)	0.004
Choledochal cyst	1 (0.7%)	1 (1.8%)	0 (0%)	0.413
Conditions related to metabolic syndrome				
Obesity	51 (37.0%)	21 (38.2%)	30 (37.0%)	1.00
T2DM	22 (15.9%)	9 (15.8%)	13 (16.0%)	1.00
Chronic liver diseases				
Cirrhosis	6 (4.3%)	5 (8.8%)	1 (1.2%)	0.081
HBV infection	16 (11.6%)	6 (10.5)	10 (12.3%)	0.482
HCV infection	7 (5.1%)	1 (1.8%)	6 (7.4%)	0.136
NAFLD	5 (3.6%)	3 (5.3%)	2 (2.5%)	0.338

BMI, body mass index; CCA, cholangiocarcinoma; HBV, hepatitis B virus; HCV, hepatitis C virus; NAFLD, non-alcoholic fatty liver disease; OV, *Opisthorchis viverrini*; SD, standard deviation; T2DM, type 2 diabetes mellitus.

Sixty (43.5%) participants were positive for *O. viverrini* antibody, which was significantly more prevalent among the case than the control group. Unhealthy lifestyles, including smoking and alcohol consumption, were found significantly more common in cases than controls, that is 23/57 (40.4%) vs. 9/81 (10.1%) for smoking and 14/57 (24.6%) vs. 1/81 (1.2%) for alcohol consumption, respectively, *P* < 0.001 for both. Similarly, CCA group had more numbers of individuals with chronic biliary tract disorders, that is, hepatolithiasis was present in 7/57 (12.3%) cases and 1/81 (1.2%) controls; and choledochal cyst was present only in 1 CCA case. There was no difference in the proportion of individuals with obesity and T2DM between the 2 groups. Regarding concurrent chronic liver diseases, the CCA group had a higher proportion of individuals with cirrhosis, that is, 5/57 (8.8%) vs. 1/81 (1.2%), *P* = 0.081, but a similar proportion of those with other chronic liver diseases to the control group. No participant in this study had PSC nor a family history of CCA.

### Factors associated with CCA development

By univariate analysis, male gender, the presence of *O. viverrini* antibody, smoking, alcohol consumption, and chronic biliary tract disorders were significantly associated with CCA (**Table 2**). By multivariate analysis, after adjusting for age and gender, chronic biliary tract disorders were the strongest independent risk factor, followed by smoking, with adjusted OR (aOR) (95% CI) of 12.7 (1.4–116.9) and 3.8 (1.3–11.8), *P* = 0.025 and 0.018, respectively. Although alcohol consumption was non-statistically significant, its *P*-value of 0.058 was marginal. Moreover, its association was strong aOR (95%CI) of 8.5 (0.9–77.6). Interestingly, the *O. viverrini* positive antibody was not independently associated with CCA (aOR [95% CI]: 1.8 [0.8–4.1], *P* = 0.148). Likewise, there was no statistically significant association between cirrhosis and CCA (aOR [95% CI]: 6.6 [0.6–77.2], *P* = 0.135).

**Table 2.** Potential variables associated with CCA

**Variables**	**Univariate analysis**		**Multivariate analysis**	
	
	**OR (95% CI)**	***P***	**aOR (95% CI)**	***P***
Age	1.0 (0.96–1.03)	0.840	1.0 (1.0–1.1)	0.589
Male gender	2.1 (1.0–4.2)	0.036	1.2 (0.5–3.0)	0.729
OV infection	2.1 (1.1–4.3)	0.031	1.8 (0.8–4.1)	0.148
Unhealthy lifestyles				
Smoking	5.4 (2.3–12.9)	<0.001	3.8 (1.3–11.8)	0.018
Alcohol consumption	26.0 (3.3–204.8)	<0.001	8.5 (0.9–77.6)	0.058
Conditions related to metabolic syndrome	1.1 (0.5–2.1)	1.00	ND	ND
Obesity	1.1 (0.5–2.1)	1.00	ND	ND
T2DM	1.0 (0.4–2.5)	1.00	ND	ND
Chronic biliary tract disorders	12.5 (1.5–98.0)	0.017	12.7 (1.4–116.9)	0.025
Hepatolithiasis	10.6 (1.3–86.8)	0.026	ND	ND
Chronic liver disease	0.8 (0.3–1.9)	0.616	ND	ND
Cirrhosis	7.7 (0.9–67.7)	0.066	6.6 (0.6–77.2)	0.135
HBV infection	0.8 (0.3–2.4)	0.743	ND	ND
HCV infection	0.2 (0.02–1.9)	0.171	ND	ND
NAFLD	2.2 (0.4–13.6)	0.398	ND	ND

aOR, adjusted odds ratio; CCA, cholangiocarcinoma; CI, confidence interval; HBV, hepatitis B virus; HCV, hepatitis C virus; NAFLD, non-alcoholic fatty liver disease; ND, not determined; OR, odds ratio; OV, *Opisthorchis viverrini*; T2DM, type 2 diabetes mellitus.

### Risk factors for each CCA subtype

Subgroup analysis using multinomial logistic regression analysis was performed to determine risk factors associated with different CCA subtypes (**Table 3**). Smoking was associated with pCCA and iCCA, with aOR (95% CI) of 7.2 (2.3–22.4) and 6.4 (2.3–17.9), *P* = 0.001 and <0.001, respectively. Alcohol consumption was found significantly associated with pCCA and iCCA subtypes, with aOR (95% CI) of 36.9 (4.1–332.1), *P* = <0.001, 28.0 (3.3–240.8), *P* = 0.002, respectively. Chronic biliary tract disorders were independently associated with iCCA and dCCA, with aOR (95% CI) of 18.2 (2.0–163.8) and 17.8 (1.5–216.0), *P* = 0.030 and 0.025, respectively. The presence of *O. viverrini* antibody and cirrhosis was not significantly associated with any CCA subtypes.

**Table 3.** Subgroup analysis of factors independently associated with CCA subtypes with reference to the control group

**Variables**	**Perihilar CCA (n = 19)**		**Intrahepatic CCA (n = 27)**		**Distal CCA (n = 11)**	

	**aOR (95% CI)**	***P***	**aOR (95% CI)**	***P***	**aOR (95% CI)**	***P***
OV infection	2.5 (0.9–6.8)	0.383	2.2 (0.9–5.4)	0.177	1.5 (0.4–5.3)	0.575
Smoking	7.2 (2.3–22.4)	0.001	6.4 (2.3–17.9)	<0.001	1.8 (0.3–9.6)	0.587
Alcohol	36.9 (4.1–332.1)	<0.001	28.0 (3.3–240.8)	0.002	8.0 (0.5–138.1)	0.390
Cirrhosis	9.4 (0.8–109.8)	0.251	10.0 (1.0–100.6)	0.178	NP	NP
Biliary tract disorder	4.4 (0.3–74.5)	0.169	18.2 (2.0–163.8)	0.030	17.8 (1.5–216.0)	0.025

aOR, adjusted odds ratio; CCA, cholangiocarcinoma; CI, confidence interval; dCCA, distal CCA; NP, no patient with cirrhosis who had distal cholangiocarcinoma; OV, *Opisthorchis viverrini*; pCCA, perihilar CCA.

**Table 4** shows the association between *O. viverrini* positivity and other CCA risks. No significant association between chronic biliary tract diseases, hepatolithiasis, or the occurrence of cirrhosis and *O. viverrini* infection was detected.

**Table 4.** Association between OV infection and other parameters

**Variables**	**OR (95% CI)**	***P*-value**
Chronic biliary tract diseases	1.0 (0.3–4.1)	0.952
Hepatolithiasis	1.3 (0.3–5.5)	0.702
Cirrhosis	0.2 (0.03–2.2)	0.208

CI, confidence interval; OR, odds ratio; OV, *Opisthorchis viverrini*.

## Discussion

Previously, almost all studies reporting a strongly positive correlation between *O. viverrini* infection and CCA were conducted in areas of extremely high CCA prevalence. In this study, participants were geographically diverse and all participants came from *O. viverrine* non-endemic areas. The prevalence of *O. viverrini* positivity in this study was 43.5%, which was lower than the reported *O. viverrini* prevalence of 67% in endemic area (the Northeast region) [1, 10,11,12]. We found that the presence of *O. viverrini* antibody was associated with the CCA risk, resembling other studies. However, its impact on CCA (OR = 2.1) was relatively lower than the previous systematic review and meta-analysis (pooled OR = 5.5–6.4) [3], and it should be noted that most studies in the systematic review were performed in an *O. viverrini* endemic area. In the adjusted analysis, *O. viverrini* infection became a statistically insignificant factor associated with CCA, with relation to other non-infectious risk factors. This might infer that *O. viverrine* infection might potentially increase the risk of CCA, but its carcinogenesis impact in the general population might not be as high as in the population in Northeastern Thailand, which might be regional-unique from both biological and environmental factors, including exposure to toxins in some local foods, for example, fermented meat and nitrate containing foods [3, 13, 14], high parasite load, as well as genetically susceptible to noxious effect from *O. viverrini* infection, for example, *GSTM1* [13, 14] or *MTHFR* polymorphisms [15].

Alcohol consumption and tobacco use were factors contributing to CCA according to studies from both *O. viverrini* endemic and non-endemic regions with varying magnitude of associations. In our study, although alcohol consumption was not statistically significant, with marginal *P*-value of 0.06, it should be observed that its magnitude of impact was one of the strongest risk factors identified in our study with aOR of 8.5 and was consistently high in all subtypes of CCA. Several large-scale studies from other non-*O. viverrini* endemic countries also suggested that alcohol consumption likely increased the CCA risk by 2–15 folds, particularly iCCA subtype [11, 16, 17]. In comparison with studies from *O. viverrini* endemic areas where alcohol consumption was found associated with CCA (pooled OR of 2.6–5.7) [14, 18], the results from our study had a higher degree of association (aOR of 8.5). These findings reiterated alcohol consumption as one of the independent contributing factors to CCA development, but its clinical impact was obscured by concurrent *O. viverrini* infection.

Previously, smoking was considered as a minor potential risk for CCA, which might be underestimated because most studies were performed with populations that concomitantly had other strong risk factors, such as *O. viverrini* infection or PSC. We found that smoking was strongly associated with CCA, but the magnitude of association was weaker than alcohol consumption with aOR of 3.8, similar to prior published study from *O. viverrine* -endemic area, showing that smoking could increase risk of CCA with the pooled OR of 1.3–2.7 [14]. However, other articles from China, South Korea, the UK, and the US showed conflicting results [11, 14, 19]. Thus, whether smoking confers a risk to CCA is still inconclusive. The carcinogenesis from tobacco use is contributed by volatile tobacco-specific nitrosamines in the mainstream smoke and carcinogenic action of nitrosamines might be significantly increased with *O. viverrini* infestation [20]. Considering that, the prevalence of CCA in our study region was still high even with a less *O. viverrini* infection rate. Genetic–environment interaction might therefore play an important role in cholangiocarcinogenesis, such as *XRCC1* and *OGG1* polymorphisms [14, 18].

Hepatolithiasis and choledochal cysts, chronic biliary tract disorders that are globally established risk factors for CCA [10, 21], have not been thoroughly investigated in Thailand [1, 3, 14]. In this study, chronic biliary tract disorders were significantly associated with CCA with the highest magnitude of relationship (aOR = 12.7). Our analysis showed that the presence of hepatolithiasis was not influenced by *O. viverrine* infestation. Accordingly, hepatolithasis from any cause confers a compelling risk for CCA. Choledochal cyst was reported to be associated with iCCA and dCCA, depending on the location of the cystic dilatation of the bile ducts [22,23,24]. When combining both diseases, chronic biliary tract disorders were thus found to be associated with both iCCA and dCCA subtypes.

In this study, cirrhosis indeed might be associated with CCA, but the association did not reach statistical significance (*P* = 0.07) likely due to the small sample size and the low proportion (3%–15%) of patients with other chronic liver diseases in our cohort. Regarding obesity, currently available information on its impact on CCA remains inconclusive [25, 26]. We did not observe a significant correlation between obesity and CCA. Although one-third of our participants were obese by Asia-Pacific Obesity Classification, their BMI were relatively lower than most Western populations, so the magnitude of obesity might not be strong enough to confer the risk to CCA in our population.

The strength of this study is the living background of the participants. We specifically designed a study population not residing in the *O. viverrini* most-endemic region, which has some regional-unique and highly influential risks to CCA. Thus, our study outcome could be more valid, representing the general population. Another strength is our diagnostic methods. The Northeastern province has an extremely high prevalence of CCA and also a high prevalence of *O. viverrini* infection; however, it was unclear whether *O. viverrini* infection was the main cause of CCA or whether it was caused by other region-specific factors. In order to confirm the association between *O. viverrini* infection and CCA, we need a high sensitivity test for detecting *O. viverrini* infection. Therefore, we diagnosed *O. viverrini* infection using serum antibody detection by ELISA technique, which has several advantages over the conventional stool test. The patients could produce antibodies to *O. viverrini* antigen as early as 2 weeks after infection, likely before the parasite started producing eggs, making this test to earlier detect *O. viverrini* infection than the stool exam. In addition, antibody detection by ELISA method is a highly sensitive technique that can detect low level of antibody in the blood. It is more beneficial than the stool exam, particularly when the parasite burden is low or intermittent.

There are limitations. First, hepatolithiasis is related to CCA and it is probably associated with chronic liver fluke infection. Although our result found a non-significant association between hepatolithiasis and *O. viverrini* infection, it might be limited by a small number of patients having hepatolithiasis. Further study with a larger number of hepatolithiasis patients may be required. Second, we diagnosed *O. viverrini* infection using serum *O. viverrini* antibody, which includes both present and past infection, not an active infection. Besides, ELISA assays may have cross-reactivity with some intestinal flukes. There are some concerns regarding diagnostic accuracy of *O. viverrini* antibody in patients with established CCA. Although there has been no study that compares diagnostic accuracy between CCA and non-CCA subjects, to the best of our knowledge, there has been no report of cross-reactivity between liver fluke antibody and CCA. This might be assumed that the test accuracy may remain highly accurate among established CCA patients. Third, some local foods such as some kinds of dishes with fermented or raw fish [3, 14], were reported to be independent risk factors for CCA. We did not collect information about diets because food information was ambiguous, subject to recall bias, and difficult to interpret. Nonetheless, these local foods were not widely available in our study region. We believe that these certain types of diets had a minimal effect on our study population. For future research, we suggest genetic testing to evaluate the susceptibility to harmful habits and dose-related carcinogenic effects.

This study highlights that in a non-genetically or environmentally prone population, harmful behaviors, especially smoking, could seriously increase the risk of CCA with effects that are even greater than *O. viverrini* infection alone and it can be said that for the general population, CCA is a lifestyle-related and preventable cancer.

## Conclusion

*O. viverrini* infection has become a trivial contribution to CCA development in non-endemic areas of liver fluke. Conversely, smoking as well as chronic biliary tract disorders are major risk factors for CCA. Therefore, attention should be raised to demonstrate that CCA is a preventable disease that requires lifestyle modifications.
